# Clinical evaluation of inline motion correction for cardiac perfusion MRI

**DOI:** 10.1186/1532-429X-13-S1-P54

**Published:** 2011-02-02

**Authors:** Aya Kino, Christopher Glielmi, Mauricio S Galizia, Andrada R Popescu, Hui Xue, Jens Guehring Guehring, Peter Weale, Sven Zuehlsdorff, James C Carr

**Affiliations:** 1 Northwestern University, Chicago, IL, USA; 2 Siemens Healthcare, Chicago, IL, USA; 3 Siemens Corporate Research, Princeton, NJ, USA

## Objective

The purpose of this study is to evaluate the impact of non-rigid registration of cardiac perfusion images on image quality and diagnostic accuracy.

## Background

Cardiac MR is often utilized to assess myocardial perfusion [1].Typically, T1 weighted images of the first pass of contrast agent are qualitatively assessed to identify hypo-enhanced regions of the myocardium. Although fully integrated approaches are available to register individual images to eliminate motion the impact on diagnostic accuracy has not been validated in patients [2].

## Materials and methods

Cardiac stress and rest first pass perfusion images (SR prepared TFL, 160×131 matrix, 2.8x2.3x10 mm spatial resolution, TI =100 ms, TE/TR =1.05/2.1 ms, TGRAPPA factor 2) were acquired in eight patients with suspected ischemic heart disease on a clinical 1.5T scanner (MAGNETOM Avanto, Siemens Healthcare). Three short axis slices were acquired during infusion of 0.075 mMol/kg of Gadolinium (Magnevist, Bayer HealthCare Pharmaceuticals, USA) at rate of 4 ml/sec. Using a conventional clinical protocol, patients were instructed to breath-hold during the beginning of the scan and resume shallow breathing after 30 hearts beats. Stress scans consisted of adenosine (Adenoscan, Astellas Pharma, USA) infusion (0.14 mg/kg/min; rate: 0.2 cc/sec; 4 min). In addition to motion correction, inline processing also consisted of temporal filtering and surface coil correction [2].Conventional and motion-corrected images were assessed by 2 radiologists using the AHA 16-segment model and scored using a four point Likert scale (1-poor/non-diagnostic; to 4-excellent without artifacts) for each slice.Signal intensity curves in each segment from both methods were normalized by baseline signal intensity of the left ventricle.

## Results

**Figure 1:**
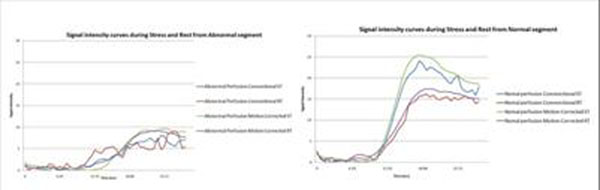
A) Time course for segment affected by surface coil correction (segment 5 AHA). B) Time course for segment less affected by this correction (segment AHA).

Eight patients were successfully scanned; perfusion defects were detected in 2 patients. The mean image quality score for motion corrected images (3.76 ± 0.34) was superior to conventional images (2.59 ± 0.46) (p<0.0001).Stress and rest normalized signal intensity curves from segments with normal and reduced perfusion are similar for both techniques but some noticeable differences are evident (Figure 1).First, temporal filtering leads to reduced high frequency fluctuations, as expected. Second, inline surface coil correction results in higher signal after contrast arrives in the myocardium for some segments (Fig.1A).

## Conclusions

Clinical evaluation of motion corrected perfusion data shows significantly higher image quality. Future work will evaluate the efficacy of inline motion correction in the context of quantitative analysis.
